# High expression of Talin-1 is associated with poor prognosis in patients with nasopharyngeal carcinoma

**DOI:** 10.1186/s12885-015-1351-5

**Published:** 2015-04-30

**Authors:** Ya-Fei Xu, Xian-Yue Ren, Ying-Qin Li, Qing-Mei He, Xin-Ran Tang, Ying Sun, Jian-Yong Shao, Wei-Hua Jia, Tie-Bang Kang, Mu-Sheng Zeng, Na Liu, Jun Ma

**Affiliations:** State Key Laboratory of Oncology in South China; Collaborative Innovation Center for Cancer Medicine, Sun Yat-sen University Cancer Center, 651 Dongfeng Road East, Guangzhou, People’s Republic of China

**Keywords:** Talin-1, Nasopharyngeal carcinoma, Prognosis, Biomarker

## Abstract

**Background:**

Talin-1 is a cytoskeletal protein that plays an important role in tumourgenesis, migration and metastasis in several malignant tumors. The aim of this study was to evaluate the expression and prognostic value of Talin-1 in nasopharyngeal carcinoma (NPC).

**Methods:**

Talin-1 mRNA and protein expression were examined in NPC cell lines and clinical nasopharyngeal tissues by quantitative RT-PCR, agarose gel electrophoresis and western blotting. The expression of Talin-1 was analyzed by immunohistochemical staining in 233 paraffin-embedded NPC specimens with clinical follow-up data and cox regression analysis was used to identify independent prognostic factors. The functional role of Talin-1 in NPC cell lines was evaluated by small interfering RNA-mediated depletion of the protein followed by the wound healing and transwell invasion assays.

**Results:**

The expression of Talin-1 was significantly upregulated in most NPC cell lines and clinical tissues at both the mRNA and protein levels. High expression of Talin-1 was significantly associated with distant metastasis (*P* = 0.001) and patient death (*P* = 0.001). In addition, high expression of Talin-1 was associated with significantly poorer overall survival (OS: HR, 2.15; 95% CI, 1.28-3.63; *P* = 0.003) and poorer distant metastasis-free survival (DMFS: HR, 2.39; 95% CI, 1.38-4.15; *P* = 0.001). Cox regression analysis indicated that high expression of Talin-1 and TNM stage were independent prognostic indicators (both *P* < 0.05). Stratified analysis demonstrated that high expression of Talin-1 was associated with significantly poorer survival in patients with advanced stage disease (stage III-IV, HR, 1.91; 95% CI, 1.09-3.35; *P* = 0.02 for OS and HR, 2.22; 95% CI, 1.24-3.99; *P* = 0.006 for DMFS). Furthermore, the depletion of Talin-1 suppressed the migratory and invasive ability of NPC cells *in vitro*.

**Conclusions:**

Our data demonstrate that high expression of Talin-1 is associated with significantly poorer OS and poorer DMFS in NPC and depletion of Talin-1 expression inhibited NPC cell migration and invasion. Talin-1 may serve as novel prognostic biomarker in NPC.

## Background

Nasopharyngeal carcinoma (NPC) is rare in most populations throughout the world, but is relatively common in Southern China and Southeast Asia where the incidence is as high as 20 to 50 per 100,000 person-years [1-4]. There were an estimated 84,400 incident cases of NPC and 51,600 NPC-related deaths in 2008 according to data from the International Agency for Research on Cancer [5]. Due to anatomic constraints and its high radiosensitivity, early-stage NPC is typically treated using radiotherapy, whereas chemoradiotherapy is the standard treatment for advanced NPC [6].

Currently, the clinical TNM (tumor, node, metastasis) staging system for NPC is most frequently used to estimate prognosis. However, large variations are observed in the clinical outcomes of patients with the same stage of disease who undergo similar therapies, indicating that the TNM staging system alone is inadequate for accurately predicting prognosis [7,8]. With progress in molecular biology, the identification of molecular biomarkers may provide additional value to prognostic predictions. In recent years, several molecular biomarkers have been identified to be associated with prognosis in NPC, such as breast cancer metastasis suppressor 1 (BRMS1), metastasis-associated protein 1 (MTA1), Dicer 11, centromere protein-H (CENP-H) and cancerous inhibitor of protein phosphatase 2A (CIP2A) [9-13]; however, prognostic prediction in NPC remains extremely poor [14]. Therefore, the discovery of novel biomarkers that could be utilized as more effective prognostic predictors would be of significant clinical value and furthermore could help to enable individualized treatment of patients with NPC.

Talin-1, a large 270 kDa cytoskeletal protein that contains 2541 amino acids, is mainly expressed in the kidney, liver, spleen, stomach, lung and vascular smooth muscle and plays an essential role in integrin activation [15]. Previous studies demonstrated that the expression of Talin-1 was highly associated with endometrioid carcinoma, oral squamous cell carcinoma (OSCC), prostate cancer and hepatocellular carcinoma [16-20]. In addition, Talin-1 can promote cancer cell adhesion, migration and invasion, and may represent a diagnostic marker of aggressive phenotypes and a potential therapeutic target for prostate cancer and OSCC [17,18]. To date, however, the expression levels and clinical significance of Talin-1 in NPC remain largely unknown.

In the present study, we examined both the mRNA and protein expression levels of Talin-1 in NPC cell lines and clinical tissue samples, and also analyzed the association between Talin-1 expression and the clinical characteristics of a cohort of patients with NPC from Guangdong Province, an area of China where the population have a high risk of NPC. Furthermore, we evaluated the prognostic value of Talin-1, in order to develop a more personalized therapy for NPC.

## Methods

### Cell lines and cell culture

Human NPC cell lines (CNE-1, CNE-2, SUNE-1, C666-1, HNE1 and HONE1) were cultured in RPMI-1640 (Invitrogen, Carlsbad, CA, USA) medium supplemented with 10% fetal bovine serum. The immortalized nasopharyngeal epithelial cell line (NP69) was cultured in keratinocyte/serum-free medium (Invitrogen) supplemented with bovine pituitary extract, as previously described [12]. All cell lines were incubated at 37°C in a 5% CO_2_ incubator.

### Patients and tissue specimens

Eight freshly-frozen NPC specimens and six normal nasopharyngeal epithelium samples were obtained from Sun Yat-sen University Cancer Center. In addition, a total of 233 pretreatment paraffin-embedded NPC specimens were collected from our hospital between January 2003 and February 2006; none of the 233 NPC patients had received radiotherapy or chemotherapy before biopsy. The clinical characteristics of all patients were recorded (Table 1). All patients were treated with conventional two-dimensional radiotherapy, and patients with stage III-IV disease also received platinum-based concurrent chemotherapy [21,22]. The median follow-up time for the entire cohort was 63.2 months (range, 5.2-91.87). The following end points (time to the first defining event) were assessed: 5-year overall survival (OS) and distant metastasis-free survival (DMFS). This study was approved by the Institutional Ethical Review Board of Sun Yat-sen University Cancer Center, and written informed consent was obtained from each patient.

**Table 1:** **Clinical characteristics of the patients with nasopharyngeal carcinoma stratified by low and high expression of Talin-1**

Characteristic	No. of patients	Expression of talin-1		*P-*value*
		Low, n (%)	High, n (%)	
**Age**				
≤45 years	114	87 (76.3)	27 (23.7)	0.09
>45 years	119	79 (66.4)	40 (33.6)	
**Sex**				
Male	169	124 (73.4)	45 (26.6)	0.24
Female	64	42 (65.6)	22 (34.4)	
**WHO type**				
I + II	8	4 (50.0)	4 (50.0)	0.23
III	225	162 (72.0)	63 (28.0)	
**VCA-IgA**				
<1:80	35	27 (77.1)	8(22.9)	0.40
≥1:80	198	139 (70.2)	59(29.8)	
**EA-IgA**				
<1:10	57	42 (73.7)	15 (26.3)	0.64
≥1:10	176	124 (70.5)	52 (29.5)	
**T Stage**				
T1-T2	113	85 (75.2)	28 (24.8)	0.19
T3-T4	120	81 (67.5)	39 (32.5)	
**N Stage**				
N0-N1	143	103 (72.0)	40 (28.0)	0.74
N2-N3	90	63 (70.0)	27 (30.0)	
**TNM stage**				
I-II	72	56 (77.8)	16 (22.2)	0.14
III-IV	161	110 (68.3)	51(31.7)	
**Locoregional failure**				
Yes	39	23 (59.0)	16 (41.0)	0.06
No	194	143 (73.7)	51 (26.3)	
**Distant metastasis**				
Yes	51	27 (52.9)	24 (47.1)	0.001
No	182	139 (76.4)	43 (23.6)	
**Death**				
Yes	57	31 (54.4)	26 (45.6)	0.001
No	176	135 (76.7)	41 (23.3)	

Abbreviations: *WHO*: World Health Organization; *VCA-IgA*: viral capsid antigen immunoglobulin A; *EA-IgA*: early antigen immunoglobulin A. P-values* were calculated using the chi-square test or Fisher’s exact test.

### RNA extraction, reverse transcription, quantitative RT-PCR and agarose gel electrophoresis

Total RNA was extracted from the cell lines using TRIzol reagent (Life Technologies, Grand Island, NY, USA). The extracted RNA (2 μg) was reverse transcribed using random primers (Promega, Madison, WI, USA) and M-MLV reverse transcriptase (Promega) for 60 min at 37°C and then 15 min at 70°C, and the product was stored at -20°C until use. Quantitative RT-PCR was performed on the PRISM 7900HT sequence detection system (Applied Biosystems, Carlsbad, CA, USA) in 15 μL reactions containing 0.5 μL of reverse transcription product, 7.5 μL of Platinum SYBR Green qPCR SuperMix-UDG reagents (Invitrogen), 1.5 μL of each PCR forward primer and reverse primer (final concentration, 2.5 μM), and 5.5 μL of ddH_2_O. The reactions were pre-incubated at 95°C for 10 min, followed by 40 cycles of denaturation at 95°C for 30 s and annealing/extension at 60°C for 1 min, then ramped from 60°C to 95°C to obtain a melting curve. The following PCR primers were used: Talin-1 forward, 5′-CTGGAGGCAACCACAGAAC-3′ and Talin-1 reverse, 5′-GTGGCTCTGGGGAACAGA-3′; glyceraldehyde-3-phosphatedehydrogenase (GAPDH) forward, 5′-CTCCTCCTGTTCGACAGTCAGC-3′ and GAPDH reverse, 5′-CCCAATACGACCAAATCCGTT -3′. All of the assays were performed in triplicate, and reactions containing either no template or no reverse transcriptase were used as negative controls. GAPDH was used as the normalization control. Agarose gel electrophoresis was performed to examine the mRNA expression level of Talin-1. PCR amplications were performed using the GoTaq Green Master Mix kit (Promega, USA) and PCR cycling conditions were as follows: 95°C for 2 min, followed by 27 cycles of 95°C for 30 s, 53°C for 30 s and 72°C for 8 s, then extension at 72°C for 5 min (Biorad, S1000 Thermal Cycler, USA). PCR products were checked by 1.5% agarose gel electrophoresis.

### Western blotting

Total proteins were extracted using sample buffer (62.5 mmol/L Tris-HCl, pH 6.8, 2% SDS, 10% glycerol, and 5% 2-β-mercaptoethanol), and the protein concentration was quantified using the Pierce® BCA Protein Assay Kit (Thermo Scientific, Rockford, IL, USA). Total proteins were separated on 6% SDS-PAGE gels and transferred onto polyvinylidene fluoride membranes (Millipore, Billerica, MA, USA). The membranes were blocked with 5% skimmed-milk powder in Tris buffered saline with tween-20 (TBS-T), incubated with mouse monoclonal anti-Talin-1 (clone 1A11) antibody (1:200; Abnova, Taipei, Taiwan), then incubated with anti-mouse IgG secondary antibody (1:5,000; Sigma, St. Louis, MO, USA). The bands were detected by enhanced chemiluminescence; α-tubulin antibody (1:5,000; Epitomics) was used as a loading control.

### Immunohistochemical staining

The expression of Talin-1 in the sections from the 233 paraffin-embedded NPC specimens was examined by immunohistochemical staining, as described previously [23]. Briefly, the tissue blocks were cut into 5 μm-thick sections, followed by deparaffinization and rehydration. The sections were microwaved for antigen retrieval in EDTA buffer, and 3% hydrogen peroxide was used to quench endogenous peroxidase activity. Non-specific binding was blocked using 1% bovine serum albumin. The sections were then incubated overnight at 4°C with mouse monoclonal anti-Talin-1 (clone 1A11) antibody (1:100; Abnova); normal goat serum was used as a negative control. After rinsing, the sections were incubated with biotinylated secondary antibody bound to a streptavidin-horseradish peroxidase complex. The bound antibody was visualized by adding 3,3-diaminobenzidine, and then the sections were counterstained with hematoxylin, dehydrated and mounted.

The sections were scored independently by two pathologists, and any disagreements were resolved by consensus. Both the proportion of positively-stained tumor cells and the intensity of staining were assessed [23,24]. The proportion of positive tumor cells was scored as follows: 1 (<10%), 2 (10-35%), 3 (35-70%), and 4 (>70%). The intensity of staining was graded on a scale from 0 to 3 as follows: 0 (no staining), 1 (weak staining, light yellow), 2 (moderate staining, yellow brown), and 3 (strong staining, brown). The staining index was calculated as the product of the staining intensity score and the proportion of positive tumor cells; using this method, staining index scores of 0, 1, 2, 3, 4, 6, 8, 9 or 12 were possible. The optimal cutoff value for high and low expression was selected based on a receiver operating characteristic (ROC) curve analysis, as previously described [9,25-27]. The best cutoff score of Talin-1 was 5. Therefore, a staining index score of < 5 was used to define NPC with low Talin-1 expression and a staining index score of > 5 was designated as high Talin-1 expression.

### Oligonucleotide transfection

CNE-2 or SUNE-1 cells were seeded into six-well plates 24 h prior to transfection. The Talin-1-silencing oligonucleotides (si*Talin-1)* or the respective controls (siCont) (GenePharma, Shanghai, China),) were transfected into the cells at a final concentration of 100 nmol/L using Lipofectamine™ 2000 (Invitrogen). The cells were harvested 48 h after transfection for the specified assays.

### Wound healing assay

For the wound healing assay, transfected CNE-2 or SUNE-1 cells were seeded into 6-well plates. After serum starvation in serum-free media for 24 h, an artificial wound was created on the confluent cell monolayer using a standard 200 μl plastic pipette tip. Cells migrated into the scratch area as single cells from the confluent sides; the width of the scratch gap was viewed under an inverted microscope and photographed at 0 h and 24 h. Three replicate wells from a six-well plate were used for this experiment.

### Transwell invasion assays

Invasion assays were performed in Transwell chambers (Corning, Steuben County, New York, USA) coated with Matrigel (BD Biosciences) on the upper surface of membrane with 8 μm-pore size. In brief, transfected CNE-2 or SUNE-1 cells were harvested, suspended in serum-free medium and 1 × 10^5^ transfected cells were plated into the upper chamber for the invasion assays, and media supplemented with 10% FBS was placed into the lower chamber. After incubation for 24 h, the cells that had invaded through the membrane to the lower surface were fixed, stained and counted under an inverted microscope. Five random fields of view were analyzed for each chamber; three independent experiments were conducted for each assay.

### Statistical analysis

The Chi-square test or Fisher’s exact test were used to analyze the relationships between Talin-1 protein expression and clinical characteristics. Talin-1 mRNA expression levels in normal nasopharyngeal epithelial tissues and NPC tissues were analyzed using the Student’s *t*-test. For functional analyses, data were presented as mean ± standard deviation and Student’s *t*-tests were used to determine the significance of the differences between two groups. The Kaplan-Meier method was used to estimate OS and DMFS, and the differences were compared using the log-rank test. A Cox proportional hazards regression analysis with backward stepwise selection was used to identify independent predictive factors for OS and DMFS and calculate hazard ratio (HR) values. The Talin-1 expression level, age, sex, clinical stage, WHO type, VCA-IgA and EA-IgA were included as covariates. All statistical analyses were performed using SPSS 16.0 software (SPSS, Chicago, IL, USA); two-tailed *P*-values < 0.05 were considered significant.

## Results

### Talin-1 is upregulated in NPC cells and tissues

**Figure 1:**
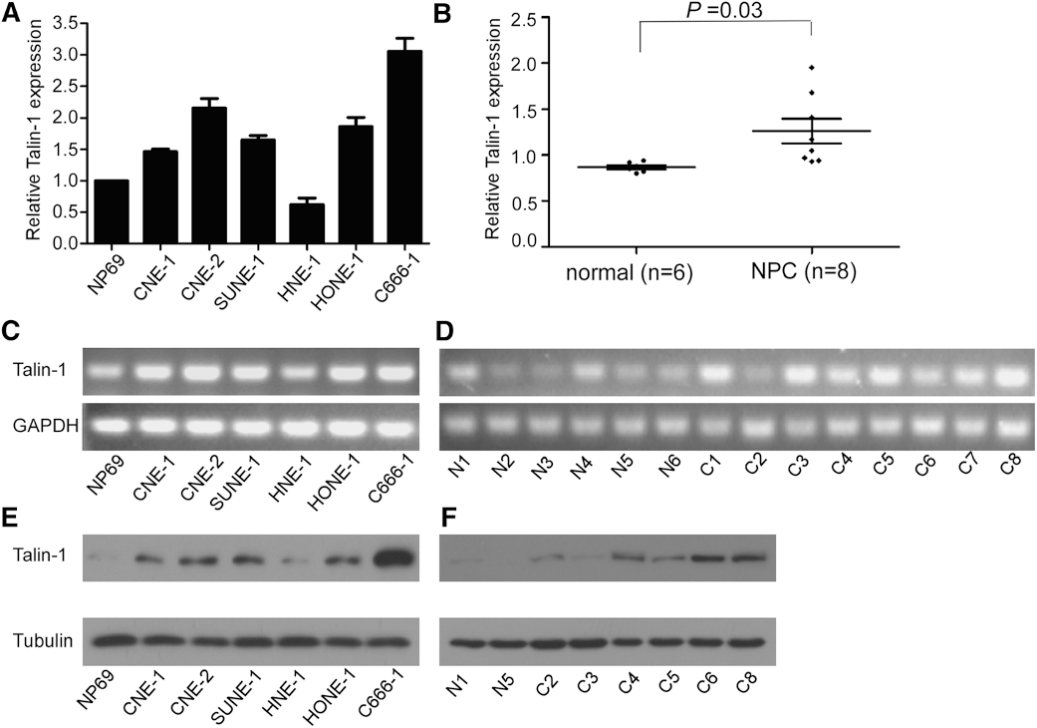
Talin-1 mRNA and protein expression in NPC cell lines and tissues. **(A and B)** Quantitative RT-PCR analysis of Talin-1 expression levels in the NP69 and NPC cell lines and in NPC (*n* = 8) and normal nasopharyngeal epithelial tissues (*n* = 6). **(C and D)** Agarose gel electrophoresis of Talin-1 expression in the NP69 and NPC cell lines and in NPC (*n* = 8) and normal nasopharyngeal epithelial tissues (*n* = 6). **(E and F)** Western blotting analysis of Talin-1 protein expression in the NP69 and NPC cell lines and in NPC and normal nasopharyngeal epithelial tissues. Data is presented as mean ± SD values; the *P*-value was calculated using the Student’s *t*-test.

The mRNA and protein expression levels of Talin-1 in the normal nasopharyngeal epithelial cell line NP69 and NPC cell lines were assessed by quantitative RT-PCR, agarose gel electrophoresis and Western blot analysis. Compared to NP69 cells, Talin-1 was significantly upregulated in all NPC cell lines tested at both the mRNA and protein levels, except for HNE1 cells (Figure 1A, C, E). Furthermore, we assessed the mRNA and protein levels of Talin-1 in eight freshly-frozen NPC tissues and six normal nasopharyngeal epithelial tissues, and observed that both Talin-1 mRNA and protein were expressed at considerably higher levels in the NPC tissues (Figure 1B, D, F). These results suggest that overexpression of Talin-1 may be involved in the progression of NPC.

### Associations between Talin-1 expression and the clinicopathological features of NPC

**Figure 2:**
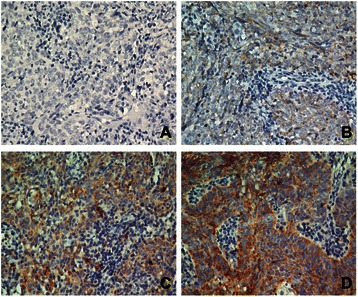
Immunohistochemical analysis of Talin-1 protein expression in clinical nasopharyngeal carcinoma tissues. Talin-1 protein expression was mainly localized to the cytoplasm of NPC cells. Representative images of **(A)** negative staining, **(B)** weak staining (light yellow), **(C)** moderate staining (yellow brown) and **(D)** strong staining (brown) in clinical NPC specimens. All images are 400 × .

To identify the associations between Talin-1 protein expression and the clinical features of patients with NPC, we analyzed Talin-1 protein expression in a set of 233 paraffin-embedded NPC tissue samples using immunohistochemistry. Representative images of Talin-1 immunohistochemical staining in NPC tissues are shown in Figure 2A-D. Positive staining for Talin-1 was mainly observed in the cytoplasm of the NPC cells. Talin-1 expression was found in 182 out of 233 (78.1%) NPCs. Using a staining index of 5 as the cutoff point in the 233 patients with NPC examined, we classified 166 (71.2%) patients with low expression of Talin-1 and 67 (28.8%) patients with high expression of Talin-1. As shown in Table 1, high Talin-1 expression was significantly associated with distant metastasis (*P* = 0.001) and patient death (*P* = 0.001). However, there were no significant associations between Talin-1 expression and other clinical features, such as age, sex, WHO type, VCA-IgA, EA-IgA, clinical stage (T stage, N stage or TNM stage) or locoregional failure (*P* > 0.05).

### High expression of Talin-1 is associated with a poor prognosis in NPC

**Figure 3:**
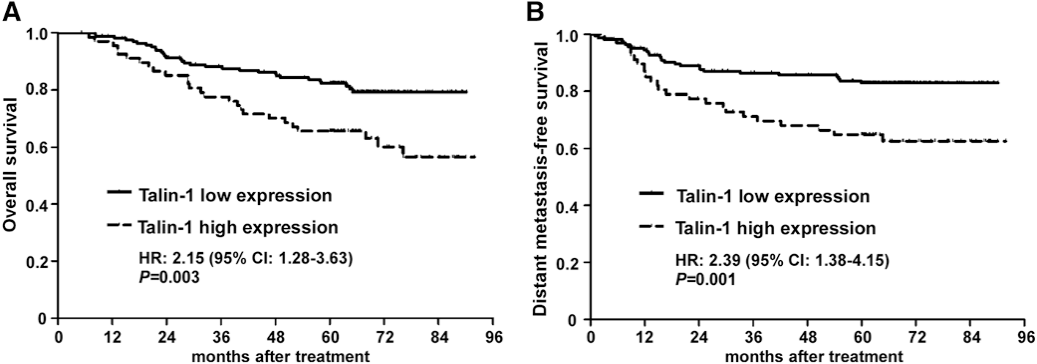
Overall and distant metastasis-free survival for patients stratified by low and high expression of Talin-1. Kaplan-Meier **(A)** overall survival and **(B)** distant metastasis-free survival curves for patients with nasopharyngeal carcinoma (*n* = 233) stratified by low and high expression of Talin-1. HR, hazard ratio, and CI, confidence interval; HR values were calculated using unadjusted Cox regression analyses; *P*-values were calculated using the log-rank test.

The relationship between Talin-1 expression and survival in NPC was assessed using Kaplan-Meier analysis and the log-rank test. As shown in Figure 3, high Talin-1 expression was associated with significantly poorer OS (HR, 2.15; 95% CI, 1.28-3.63; *P* = 0.003) and poorer DMFS (HR, 2.39; 95% CI, 1.38-4.15; *P* = 0.001; Figure 3A-B). The cumulative 5-year survival rate for the low Talin-1 expression group was 78.8% (95% CI, 75.0-82.5) compared to only 68.4% (95% CI, 61.0-75.8) for the high Talin-1 expression group.

**Figure 4:**
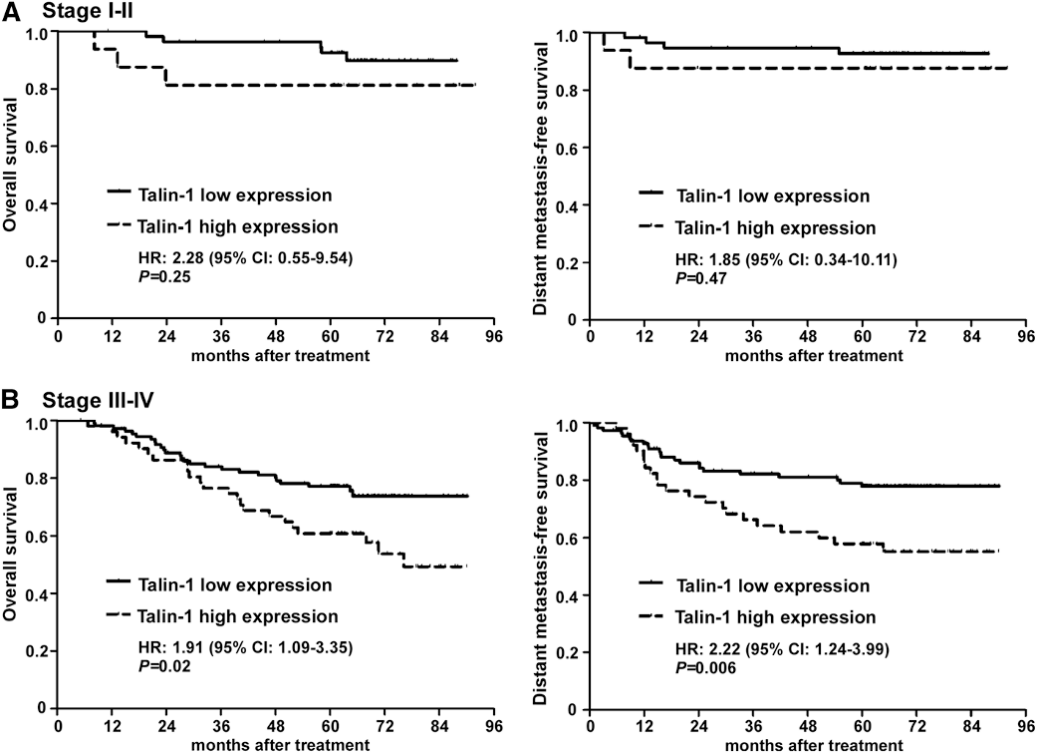
Upregulation of Talin-1 is associated with poor survival in advanced nasopharyngeal carcinoma. Overall and distant metastasis-free survival curves for patients with **(A)** Stage I-II (*n* = 72) and **(B)** Stage III-IV (*n* = 161) nasopharyngeal carcinoma. Hazard ratio (HR) and confidence interval (CI) values were calculated using unadjusted Cox regression analyses; *P*-values were calculated using the log-rank test.

Furthermore, stratified analysis indicated that in the subgroup with early-stage NPC (stage I-II, *n* = 72), the differences in OS and DMFS between patients with high or low Talin-1 expression were not significant (*P* = 0.25 and *P* = 0.47, respectively; Figure 4A). However, in the advanced disease subgroup (stage III-IV, *n* = 161), patients with high Talin-1 expression had significantly poorer OS (HR, 1.91; 95% CI, 1.09-3.35; *P* = 0.02) and poorer DMFS (HR, 2.22; 95% CI, 1.24-3.99; *P* = 0.006) than those with low Talin-1 expression (Figure 4B). These results suggest that high expression of Talin-1 is associated with a poorer prognosis in NPC, especially in patients with advanced disease (stage III-IV).

### High expression of Talin-1 is an independent prognostic factor in NPC

Univariate and multivariate Cox regression analyses were carried out to investigate the effect of different clinical variables, including Talin-1 expression, TNM stage, age, sex, WHO type, VCA-IgA and EA-IgA, on OS and DMFS. As shown in Table 2, univariate analyses indicated that high Talin-1 expression was associated with significantly poorer OS and poorer DMFS (*P* = 0.004 and 0.002, respectively) compared to low Talin-1 expression. In addition, advanced stage disease (stage III-IV) was associated with significantly poorer OS and poorer DMFS (*P* = 0.003 and 0.002, respectively) compared to early stage disease (stage I-II). However, age, sex, WHO type, VCA-IgA and EA-IgA had no significant impact on OS or DMFS (all *P* > 0.05). Furthermore, multivariate analyses confirmed that high expression of Talin-1 and TNM stage were independent prognostic indicators associated with OS and DMFS in NPC (both *P* < 0.05).

**Table 2:** **Univariate and multivariate cox regression analyses of prognostic factors in nasopharyngeal carcinoma**

	Univariate analysis			Multivariate analysis		
Variable	HR	95% CI	*P-*value*	HR	95% CI	*P-*value*
**Overall survival**						
Talin-1 expression (high vs. low)	2.15	1.28-3.63	0.004	1.87	1.10-3.16	0.02
TNM stage (III-IV vs. I-II)	3.06	1.45-6.47	0.003	2.97	1.40-6.29	0.005
Age (≥45 years vs. <45 years)	1.63	0.96-2.78	0.07			
Sex (male vs. female)	1.57	0.83-2.96	0.17			
WHO Type (III vs. I-II)	0.63	0.20-2.03	0.44			
VCA-IgA (≥1:80 vs. < 1:80)	1.50	0.64-3.49	0.35			
EA-IgA (≥1:10 vs. < 1:10)	1.04	0.56-1.93	0.90			
**Distant metastasis-free survival**						
Talin-1 expression (high vs. low)	2.39	1.38-4.15	0.002	2.18	1.25-3.78	0.006
TNM stage (III-IV vs. I-II)	3.78	1.61-8.86	0.002	3.48	1.48-8.17	0.004
Age (≥45 years vs. < 45 years)	1.58	0.90-2.78	0.11			
Sex (male vs. female)	1.51	0.77-2.94	0.23			
WHO Type (III vs. I-II)	0.54	0.17-1.73	0.30			
VCA-IgA (≥1:80 vs. < 1:80)	1.10	0.50-2.45	0.81			
EA-IgA (≥1:10 vs. < 1:10)	1.30	0.65-2.60	0.45			

Abbreviations: *WHO*: World Health Organization; *VCA-IgA*: viral capsid antigen immunoglobulin A; *EA-IgA*: early antigen immunoglobulin A. P-values* were calculated using univariate and multivariate cox regression analyses.

### Talin-1 depletion has significant effect on the NPC cell migration and invasion in vitro

**Figure 5:**
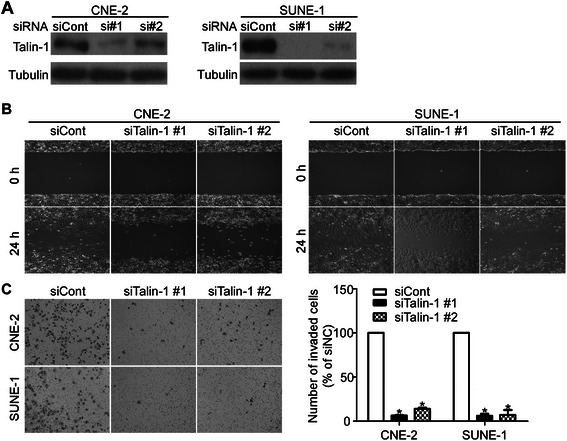
Talin-1 depletion had significant effect on the NPC cell migration and invasion in vitro. **(A)** Talin-1 protein expression by western blotting after transfection with siTalin-1 or siCont. **(B)** Representative images of the wound healing assay of CNE-2 and SUNE-1 cells transfected with siTalin-1 or siCont. **(C)** Representative images (left) and quantification (right) of the Transwell invasion assay of CNE-2 and SUNE-1 cells transfected with siTalin-1 or siCont. Values are mean ± SD; *P < 0.01.

To investigate the effects of aberrant expression of Talin-1 on the migratory and invasive ability of NPC cells, CNE-2 and SUNE-1 cells were transfected with siTalin-1 or siCont. Figures 5A showed that Talin-1 protein levels were significantly reduced by transfection of siTalin-1. The wound healing assay demonstrated that the migratory ability of CNE-2 and SUNE-1 cells transfected with siTalin-1 was significantly lower than those of cells transfected with siCont (Figure 5B). In addition, the Transwell invasion assay showed that the invasive ability of NPC cells transfected with siTalin-1 was significantly lower than those of cells transfected with siCont (Figure 5C; ^*^P < 0.01). These results indicate that Talin-1 depletion significantly reduces the migratory and invasive ability of NPC cells *in vitro*.

## Discussion

The clinical prognosis of patients with NPC has improved significantly with recent advances in diagnostic technologies and therapeutic interventions. However, patients with the same clinical stage of NPC still have varying clinical outcomes when receiving identical therapeutic interventions. Therefore, it is particularly important to identify novel biomarkers that could more effectively predict prognosis and further help to achieve individualized treatment of patients with NPC. The present study demonstrates that Talin-1 is upregulated in NPC at both the mRNA and protein levels, and that high expression of Talin-1 is associated with a significantly poorer prognosis in patients with NPC. In addition, Talin-1 was identified as an independent prognostic indicator in multivariate analysis, indicating that Talin-1 may serve as a novel prognostic biomarker to guide clinical practice and research on NPC.

Talin-1 is a cytoskeletal protein that acts as a key adaptor protein to regulate integrin conformation and cell migration, and has been shown to play an important role in promoting tumor cell adhesion, migration and invasion in different types of cancer. Previous studies showed that high expression of Talin-1 was associated with a poor clinical prognosis in OSCC [17,28]. In addition, Sakamoto et al. reported that Talin-1 was significantly upregulated in primary tumors and metastatic prostate cancer compared with the normal prostate gland [18]. Furthermore, higher expression of Talin-1 was observed in triple-negative breast cancer cell lines, and low Talin-1 expression was a prognostic marker for better outcome after cytotoxic chemotherapy [29]. Together, these studies indicate that Talin-1 may serve as a potential diagnostic and prognostic marker of aggressive phenotypes and may represent a potential therapeutic target for various types of cancer.

In this study, the Talin-1 expression was found in 182 out of 233 (78.1%) NPCs and the percent of Talin-1 overexpression was 28.8%. Lai MT et al. revealed that the Talin-1 expression was found in 101 out of 163 (62%) oral cavity cancer and the percent of Talin-1 overexpression was 19.6% [17]. The above results showed that the expression of Talin-1 were higher in NPC than that in oral cavity cancer. However, Talin-1 expression in hepatocellular carcinoma (HCC) was controversial. For example, Kanamori et al. found that Talin-1 was up-regulated in HCC (60.4%) [20]. On the other hand, Zhang et al. proved that Talin-1 was down-regulated in HCC liver tissues [19]. Therefore, we think that the expression of Talin-1 has tissue-specificity and further studies are needed to explore the role of Talin-1 in different cancers. In addition, we observed that high Talin-1 expression was significantly associated with distant metastasis and patient death, but not with locoregional failure. These results suggest that overexpression of Talin-1 may be involved in the progression of NPC.

It is well-recognized that local control of NPC improved significantly with the advent of intensity-modulated radiotherapy, and that the majority of treatment failures and deaths in patients with NPC are currently attributable to distant metastasis [30]. Therefore, it is vital to detect metastasis-associated biomarkers that can effectively distinguish patients with NPC who are at a high risk of metastasis at the time of diagnosis. Integrin signaling plays a crucial role in cell invasion and migration, and a variety of extracellular matrix (ECM)-remodeling factors that may contribute to these processes have been identified [31-33]. As a focal adhesion complex protein that regulates integrin interactions with the ECM, Talin-1 has been identified as a novel regulator of the invasive and metastatic potential of tumor cells, as overexpression of Talin-1 significantly enhanced the migration and invasion of prostate cancer and OSCC cells *in vitro,* and was associated with advanced tumour stage and poorer clinical outcome [17,18]. In the present study, the wound healing assay and the transwell invasion assay showed that Talin-1 depletion could significantly reduce the migratory and invasive ability of NPC cells *in vitro* and survival analysis demonstrated that high expression of Talin-1 was associated with significantly poorer OS and DMFS in patients with NPC. Further stratified analysis revealed that high expression of Talin-1 was associated with significantly poorer survival in patients with stage III-IV disease. More importantly, multivariate analysis showed that high expression of Talin-1 was an independent prognostic factor in NPC. These results suggests that Talin-1 may be a useful prognostic biomarker for NPC. However, further studies are needed to confirm these findings in other cohorts of patients with NPC.

The mechanism of action of Talin-1 during the development and progression of cancer is poorly characterized and may be complex. Talin-1 may trigger a conformational change in the extracellular domains of β-integrin, which may increase the affinity of β-integrin for ECM proteins by linking the cytoplasmic domains of integrin β subunits to actin filaments [34,35]. Previous studies had indicated that the activation of the FAK/AKT pathway was associated with increased proliferation, migration and invasion in a variety of tumors [36-39]. Recently, Sakamoto and colleagues found that overexpression of Talin-1 enhanced prostate cancer cell adhesion, migration and invasion by stimulating FAK, Src and GSK3β independently of integrin signaling and also conferred resistance to anoikis [18]. Other researchers reported that inhibition of the binding of Talin-1 to integrin could prevent integrin activation and downregulate downstream oncogenic signaling *in vitro* [33,40].

To date, the mechanisms leading to the high expression of Talin-1 in human cancers are not very clear. Tang et al. found that TLN1 was overexpressed and associated with aggressiveness and metastasis in ovarian serous carcinoma and microRNA-9 could inhibit Talin-1 expression by targeting its 3′untranslated region and further led to the inhibition of the FAK/AKT pathway [41]. The current study indicates that Talin-1 plays an important role in the development and progression of NPC. Further investigation of the function and mechanism of action of Talin-1 may provide new opportunities for therapeutic targeting of NPC and we will further explore the precise mechanisms by which Talin-1 mediates progression and metastasis in NPC in a future study.

## Conclusions

Talin-1 is upregulated at both the mRNA and protein levels in NPC. High expression of Talin-1 was significantly associated with poorer OS and DMFS in NPC, especially in patients with advanced stage disease (stage III-IV). Talin-1 may have potential as a novel prognostic biomarker and potential therapeutic target in NPC.

## Abbreviations

NPC: Nasopharyngeal carcinoma
IHC: Immunohistochemistry
WHO: World Health Organization
VCA-IgA: Viral capsid antigen immunoglobulin A
EA-IgA: Early antigen immunoglobulin A. OS, overall survival
DMFS: Distant metastasis-free survival
HR: Hazard ratio
CI: Confidence interval
OSCC: Oral squamous cell carcinoma
ECM: Extracellular matrix
